# Analysis of *Litopenaeus vannamei* Transcriptome Using the Next-Generation DNA Sequencing Technique

**DOI:** 10.1371/journal.pone.0047442

**Published:** 2012-10-11

**Authors:** Chaozheng Li, Shaoping Weng, Yonggui Chen, Xiaoqiang Yu, Ling Lü, Haiqing Zhang, Jianguo He, Xiaopeng Xu

**Affiliations:** 1 MOE Key Laboratory of Aquatic Product Safety/State Key Laboratory for Biocontrol, School of Life Sciences, Sun Yat-sen University, Guangzhou, People’s Republic of China; 2 School of Marine Sciences, Sun Yat-sen University, Guangzhou, People’s Republic of China; 3 Division of Cell Biology and Biophysics, School of Biological Science, University of Missouri-Kansas City, Kansas City, United States of America; Biodiversity Insitute of Ontario - University of Guelph, Canada

## Abstract

**Background:**

Pacific white shrimp (*Litopenaeus vannamei*), the major species of farmed shrimps in the world, has been attracting extensive studies, which require more and more genome background knowledge. The now available transcriptome data of *L. vannamei* are insufficient for research requirements, and have not been adequately assembled and annotated.

**Methodology/Principal Findings:**

This is the first study that used a next-generation high-throughput DNA sequencing technique, the Solexa/Illumina GA II method, to analyze the transcriptome from whole bodies of *L. vannamei* larvae. More than 2.4 Gb of raw data were generated, and 109,169 unigenes with a mean length of 396 bp were assembled using the SOAP denovo software. 73,505 unigenes (>200 bp) with good quality sequences were selected and subjected to annotation analysis, among which 37.80% can be matched in NCBI Nr database, 37.3% matched in Swissprot, and 44.1% matched in TrEMBL. Using BLAST and BLAST2Go softwares, 11,153 unigenes were classified into 25 Clusters of Orthologous Groups of proteins (COG) categories, 8171 unigenes were assigned into 51 Gene ontology (GO) functional groups, and 18,154 unigenes were divided into 220 Kyoto Encyclopedia of Genes and Genomes (KEGG) pathways. To primarily verify part of the results of assembly and annotations, 12 assembled unigenes that are homologous to many embryo development-related genes were chosen and subjected to RT-PCR for electrophoresis and Sanger sequencing analyses, and to real-time PCR for expression profile analyses during embryo development.

**Conclusions/Significance:**

The *L. vannamei* transcriptome analyzed using the next-generation sequencing technique enriches the information of *L. vannamei* genes, which will facilitate our understanding of the genome background of crustaceans, and promote the studies on *L. vannamei.*

## Introduction

Pacific white shrimp, *Litopenaeus vannamei*, formerly *Penaeus vannamei*, belongs to the Penaeidae family of decapod crustaceans, is the major species of farmed shrimps in the world [1]. Because of its great economic value and important evolutionary status, more and more studies have been focused on breeding, growth, development, immunity, genetics and evolution of *L. vannamei* in recent twenty years [2]–[6]. The *L. vannamei* genome with higher recombination rates than other genomes of closely related penaeid prawns is predicted to be 2.0 Gigabases and has not been sequenced up to now [7], [8]. The absence of a fully sequenced and assembled genome hindered the studies on *L. vannamei*, including determination of gene functions and regulations, and establishment of novel genetic manipulation technologies.

Many transcriptome studies of *L. vannamei* have been carried out and a large number of expressed sequence tags (ESTs) were obtained using cDNA library and Sanger sequencing methods. By May 2012, 162,993 ESTs from many organs and tissues have been released on Genbank. These data have been used for cloning functional genes, selecting genetic molecular markers, and designing cDNA microarrays [2], [9]–[11]. However, because of the limitations of the traditional methods used for ESTs sequencing, the now available transcriptome data of *L. vannamei* are still insufficient for research requirements relative to the size of its genome. Moreover, the now available EST sequences have not been systematically analyzed. Up to now, only 7968 unigenes have been assembled and annotated, largely limiting the use of the ESTs sequence data.

**Figure 1:**
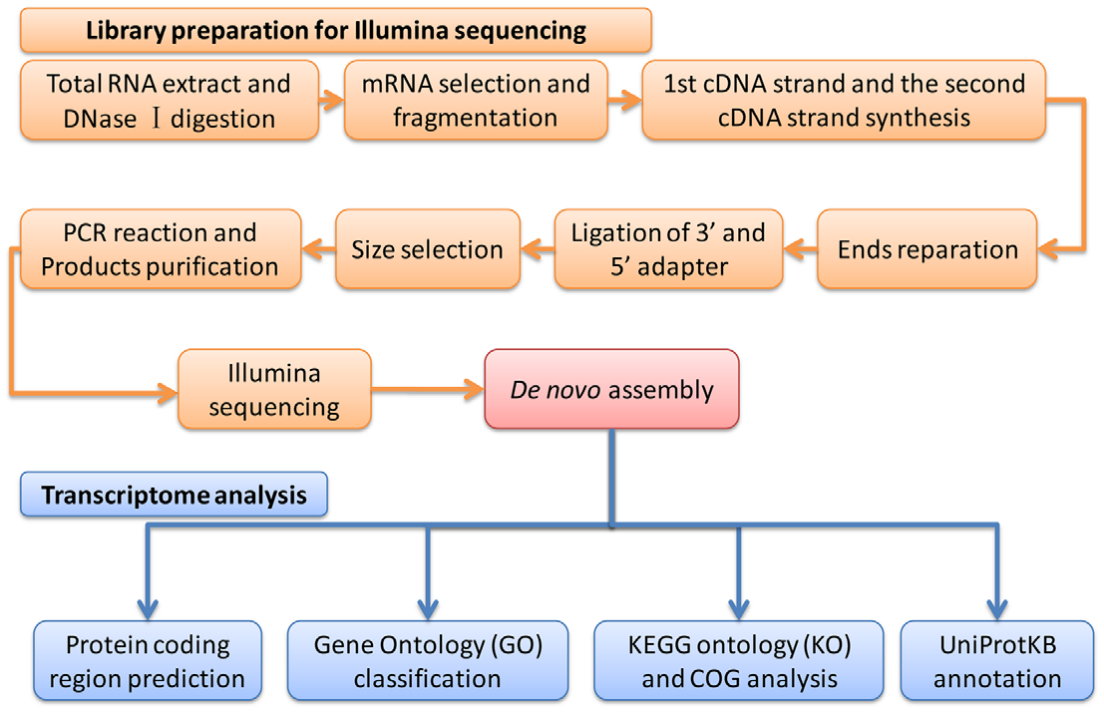
Workflow diagram for transcriptome sequencing, assembly and analysis.

The next-generation high-throughput DNA sequencing techniques, such as Solexa/Illumina (Illumina), 454 (Roche) and SOLiD (ABI), have been developed and growing rapidly in recent years [12]. They can sequence millions of DNA fragments simultaneously and provide Gigabases of data with high fidelity in a single machine run, greatly improving work efficiency and increasing data output [13]. The enormous advantages of these technologies make them admirably suited for genomics research, such as *de novo* and re-sequencing of genome, mRNA and microRNA [14]–[18]. Especially in transcriptome analysis, the usage of the next-generation sequencing techniques make it no longer necessary to establish cDNA libraries with bacteria cells as carriers, which could introduce DNA fragments deletion during the cloning process [19].

In this study, we analyzed the transcriptome of whole bodies of *L. vannamei* larvae using Solexa/Illumina high-throughout sequencing method, providing over 2.4 Gb data of raw sequences, which were assembled into 109,169 unigenes. We further annotated the unigenes by matching against Nr, Swissprot, Clusters of Orthologous Groups of proteins (COG), Gene ontology (GO), and Kyoto Encyclopedia of Genes and Genomes (KEGG) databases. Part of the results of unigene assemblies and annotations were primarily verified by RT-PCR, gel electrophoresis, Sanger sequencing, and real-time PCR. The assembled and annotated unigenes provide useful information for the studies on genomes and functional genes of *L. vannamei* and crustaceans.

## Methods

### Breeding of L. vannamei

Newly hatched *L. vannamei* larvae from a relatively high-WSSV-resistant family [20], obtained from Hengxing (Evergreen) shrimp farm in Zhanjiang, China, were fed with larvae of *Chirocephalus diaphanus* and bred in seawater of pH 8.0 and 30 g kg^−1^ salinity in a 3 m^3^ indoor tank with recirculating and filtering units at 28°C.

**Table 1:** Analyzed genes and their specific primers.

Unigene	similarity	Size (bp)	RT-PCR Primer sequence (5′–3′)	Realtime-PCR Primer sequence (5′–3′)
**98112**	abdominal-A[*Strigamia maritima*]	586	F: TCGTCGTGGACAGCCGTTTGR: CGGTTCTCGTGCCTGGTGTC	F: CGTCTGTGGTCGCCGTCATR: CAGAAGTATGTACCCCTACGTGAGT
**10296**	abdominal-B(*Strigamia maritime*)	1049	F: GGCTAGGGGCAGAGGGGTGACAGGAR: CAGCGAGGGAGGAGGACTAGACAG	F: GGCATGACTCCGTGGTCATCR: CCACGTACTATAACATTGCGGAC
**54158**	homeotic antennapedia protein [*Culex quinquefasciatus*]	216	F: CAGCTCGAGGGTCTGGTACCGCGTGR: GGAGGCGAAGGAGCCGAAG	F: TGGTACCGCGTGTAGGACGTTCR: CTCAGATCTACCCGTGGATGAAGAG
**16317**	Wnt6 [*Monodelphis domestica*]	555	F: CCATCGTGATGCTCCACAACAACR: GCTATCATCAATTATTCCGTAATC	F: ATGTCGCTTCAAGTTCTGCTGTGR: CCGGACGCTATCATCAATTATTCC
**18019**	Wnt10 [*Tribolium castaneum*]	408	F:CTGGCACCGAACCGGGTGTTGTCGR:TCTGGGACCACGTGCTCGACCC	F: GTGATGGCGTGACCGAAGGR: CGGAAGGCAGTTTGCGAATC
**105360**	beta-catenin [*Parhyale hawaiensis*]	979	F:GACTTTGGTTGCAGCATCTGAGAGGR: CAACCAGGCTGCCTTCACCAGC	F: GCAGCAGGAGGTTGTGGAGTGR: CGCCAGGGTTTATTAGCCATTTTC
**92779**	Pumilio(*Apis mellifera*)	468	F: GTTTTGTCGTCATCGAGGCCGTR: AAAAGCTGTGGGGAGTGGAAG	F: CTGAGTTGCAGGTTGGCCATGR: TCAAGATGGTCGAGTACGTGTTG
**99210**	pumilio homolog 2-like (*Saccoglossus kowalevskii*)	619	F: CTGGCGGATCCTCATCCTTATTCR: GCTGAATAACACGGCAACCATAGG	F: GGCCACGTTTTGCCTTTAGCR: ATCCAGTTCACGCACGATGTCT
**95206**	Dorsal (*Litopenaeus vannamei*)	514	F: AGTTACGAGAGGAGATTAGAGTGGR: AGTCTAGAGGCAAATACTGGAATG	F: TTCCAGACCGGGTTTTCTCATCR: TTCCCCTTTTCTGATCCCTCG
**99694**	Spalt (*Tribolium castaneum*)	635	F: GCTGTCCCCCGAGGCCCTCAGR: TTGAGAAGCCTCGGTCGCAGATG	F: CCCACGCCGTCGGACTACTR: GACGGAGCCGCTGATATTGTG
**10400**	extra sex combs (*Schistocerca americana*)	567	F: GCATTTTGTTGGTCATGGAAATGCR: TCCAAAGCAAACCTCATAAACCAG	F: TTTCAACCCAAGCAGAGCAGTGR: CCAACGCACACAATCCACATAGT
**20337**	HIRA (*Takifugu rubripes*)	4036	F: CAAAGATGGCGGAGGAAGCGTCR: GCTTGCTAACTTTCATGAGTTTCAC	F: GGAGGCTTGGCTTTTGTTAGGTR: CACTTTGATGACAGGGAGGCAG

### Illumina Sequencing

One hundred *L. vannamei* larvae at 20 days post spawning were fasted for 24h to avoid contamination from the feed *Chirocephalus diaphanous* larvae and then sampled. Total RNA was extracted using the SV Total RNA Isolation System (Promega) according to the manufacturer’s protocol. The total RNA concentration, quality and integrity were determined using a NanoDrop 1000 spectrophotometer and an Agilent 2100 Bioanalyzer. The total RNA with absorbance 260/280 nm Ratio of ∼2.0 was chosen and treated with DNase I prior to library construction. Poly-(A) mRNA was then purified using oligo(dT) magnetic beads and fragmented by treatment with divalent cations and heat, followed by reverse-transcribing into cDNA using reverse transcriptase and random hexamer-primers. The second-strand cDNA was synthesized using RNaseH and DNA polymerase I. The double-stranded cDNA was end-repaired using T4 DNA polymerase, Klenow fragment, and T4 polynucleotide kinase followed by a single (A) base addition using Klenow 3′ to 5′ exo-polymerase, and further ligated with an Illumina PE adapter oligo mix using T4 DNA ligase. Adaptor-modified fragments with length of 200±25 bp were selected by gel purification and subjected to PCR amplification as templates. After 15 cycles of PCR amplification the libraries were sequenced on an Illumina sequencing platform (GAII) and the raw reads were generated using Solexa GA pipeline 1.6.

**Table 2:** Summary statistics of *L. vannamei* transcriptome sequencing and assembly.

Summary	Number of total nucleotides(nt)	Number of mean length of total reads	Number of mean length of total reads(bp)
Output statistics of sequencing	2,465,545,140	27,394,946	90
Assembly	Contig	Scaffold	Unigene
Length distribution			
75–99	702,730	−	−
100–499	159585	139,764	86,564
500–999	15098	16,271	16,284
1000–1499	3,331	3,993	4,007
1500–1999	992	1,311	1,308
≥2000	663	1,003	1,006
Total No.	882,399	162,342	109,169
Length statistics(bp)			
Mean length	127	306	396
N50	90	399	478
Total length(Mb)	112	49.6	43.2

**Figure 2:**
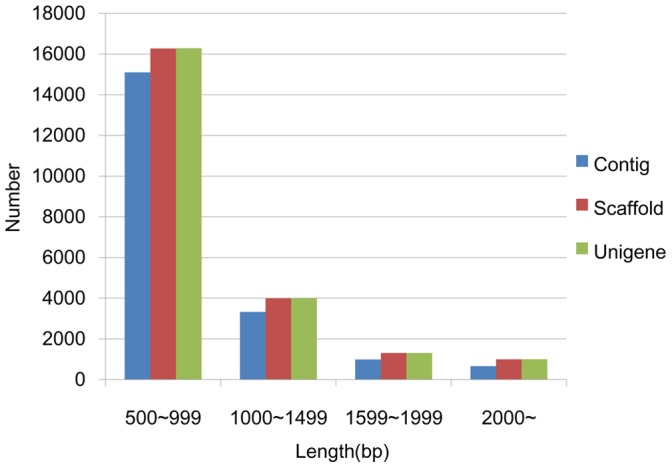
The size distributions (>500 bp) of Contigs, Scaffolds, and Unigenes.

### Transcriptome *De novo* Assembly and Analysis

Fig. 1 shows the workflow of transcriptome assembly and analysis. The raw reads of Illumina sequencing were preprocessed by removing adaptor sequences, low-quality reads (reads with ambiguous bases N), and duplication sequences, and were then assembled using the SOAP denovo software (http://soap.genomics.org.cn/soapdenovo.html) with the default settings. Firstly, the clean reads are combined by SOAP denovo based on sequence overlap to form longer fragments without N, which are called contigs, and then the reads are mapped back to contigs. Next, scaffolds were made using SOAP denovo by connecting the contigs with N to present unknown sequences between each two contigs in a same transcript. Scaffolds’ gaps can be filled by paired-end reads of sequencing to get sequences with least Ns and cannot be extended on either end. Such sequences are defined as unigenes, and the following analysis are based on them [15], [21], [22].

After ruling out short-length sequences and low-quality sequences containing more than 10% ambiguous ‘N’ nucleotides or consecutive 14 ‘N’ nucleotides, unigenes with a minimum length of 200 bp were selected, submitted to NCBI Transcriptome Shotgun Assembly (TSA) database (http://www.ncbi.nlm.nih.gov/genbank/TSA.html, accession number: JP355723-JP376614 and JP382831-JP435443), and subjected to annotation analysis. The coding regions (CDSs) of the assembled >200 bp unigenes were determined by matching sequences against NCBI Nr (non-redundant, http://www.ncbi.nlm.nih.gov/), UniProtKB (including Swiss-prot and TrEMBL, http://www.uniprot.org/), KEGG, and COG databases in turn using the BLASTx algorithm of the BLAST software (ftp://ftp.ncbi.nih.gov/blast/executables/release/2.2.23/) with a significant threshold of E value<0.00001. Unigenes (>200 bp) that cannot be aligned to Nr and Swiss-prot databases were scanned by ESTScan (http://www.ch.embnet.org/software/ESTScan2.html) to determine their CDSs [23].

**Figure 3:**
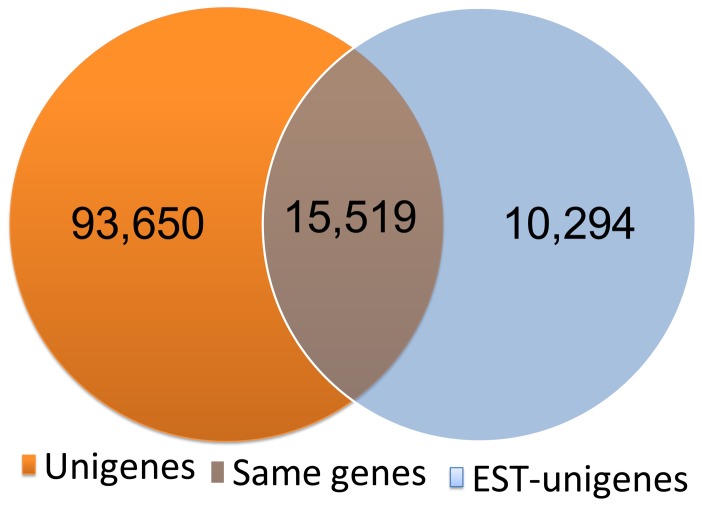
Venn chart for comparisons between assembled transcriptome unigenes and assembled EST-unigenes.

To compare the assembled unigenes with *L.vannamei* ESTs available from Genbank (http://www.ncbi.nlm.nih.gov/nucest/?term=Litopenaeus vannamei), EST sequences were firstly clustered by TIGR Gene Indices clustering tools (TGICL) [24] with minimum overlap length of 60 bp, minimum identity of 94% for the overlap, and maximum mismatched overhang of 30 bp, and then combined using Phrap (http://bozeman.mbt.washington.edu/phrap.docs/phrap.html) with minmatch 35, minscore 35 and repeat stringency 0.95. Comparison between assembled unigenes and assembled ESTs was performed using BLASTn algorithm and E-value threshold of 0.00001.

Based on Nr annotation, we used BLAST2GO program (http://www.BLAST2go.org/) to get GO annotation of unigenes [25]. GO functional classification for all unigenes was performed using WEGO software (http://wego.genomics.org.cn/cgi-bin/wego/index.pl) [26]. KEGG metabolic pathway annotation and COG classification of unigenes were determined by BLASTx searching against KEGG database and COG database, respectively [27]–[30].

### RT-PCR and Real-time PCR Assays

12 annotated unigenes that may relate to embryo development were selected to be analyzed using real-time PCR and RT-PCR, and their specific primers were listed in Table 1. For real-time PCR assays, Parent prawns mated and laid eggs in a 3 m^3^ tank at 28°C with seawater of pH 8.0 and 30 g kg^−1^ salinity. The timing of sampling was according to the embryogenesis stages, which were determined by microscopic examination and reference to previous studies [31], [32]. 60 fertilized eggs per sample were collected at 0, 140, 215, 275, 480, 600 and 660 minutes post-spawning (mps), respectively. The spawning and sampling experiments were repeated for three biological replicates. Total RNA of each sample was isolated with TRIzol (Invitrogen, USA) and subjected to DNase I treatment (Promega, USA) according to the manufacturer’s protocols. The cDNA was synthesized with SuperScript II RT (Invitrogen, USA), and quantitative real-time PCR was triply performed using the LightCycle 480 System (Roche, Germany) with a volume of 10 µl contained 1 µl of 1∶10 cDNA diluted with ddH_2_O, 5 µl of 2×SYBRGreen Master Mix (Toyobo, Japan), and 250 nM of each primer. The cycling parameters were 95°C for 2 min to activate the polymerase, followed by 40 cycles of 95°C for 15 s, 62°C for 1 min, and 70°C for 1 s. Cycling ended at 95°C with 5°C/s calefactive velocity to create the melting curve. Fluorescence measurements were taken at 70°C for 1 s during each cycle. Expression levels of each gene were normalized to 18S ribosomal RNA (18S rRNA, GenBank accession number: AF186250, 18S-rRNA-qF and 18S-rRNA-qR primers: 5′-CTGCGACGCTAGAGGTGAAATTC-3′ and 5′- AGGTTAGAACTAGGGCGGTATCTG-3′). Data were calculated with the relative quantification method described by Muller *et al.* [33], and subjected to statistical analysis.

For RT-PCR assays, the 12 unigenes were amplified by LA Taq DNA polymerase (TaKaRa, Japan) using cDNA from *L. vannamei* larvae at 20 days as template. The PCR products were analyzed using agarose gel electrophoresis, and subcloned into the PMD19-T vector (TaKaRa, Japan) for Sanger sequencing.

**Table 3:** Summary statistics of *L. vannamei* transcriptome blast assignment.

Species	Number of Unigenes	Number of Nr annotations	Number of Swiss-prot annotations	Number of TrEMBL annotations	ESTscan prediction	Number of COG hits	Number of GO mapped	Number of KEGG hits
*Litopenaeus vannamei*	109,169	27,789	27,424	32,439	11,886	11,153	45,601	18,154

## Results

### Illumina Sequencing and Sequence Assembly

Sequences of mRNA pooled from one hundred whole bodies of *L. vannamei* larvae were analyzed on an Illumina GAII platform. Up to 27,394,946 reads (accumulated length of 2,465,545,140 bp with a GC percentage of 47.89%) were obtained and assembled into 882,339 contigs (>75 bp, mean length of 127 bp and an N50 length of 90 bp), of which 79.64% (702,760) were with length of 75–100 bp, 11.34% (100,065) with 100–200 bp, 3.93% (34,655) with 200–300 bp, 1.80% (15,914) with 300–400 bp, 1.01% (8,951) with 400–500 bp, and 2.27% (20,054) with >500 bp. A total of 162,342 scaffolds (>100 bp), with a mean length of 306 bp and an N50 of 399 bp, were generated by assembling and paired end-joining of these contigs. About 86.09% (139,764) of scaffolds were 100–500 bp long, 10.02% (16,271) were 500–1000 bp, 2.46% (3,993) were 1000–1500 bp, 0.81% (1,311) were 1500–2000 bp, and 0.62% (1,003) were >2000 bp. Most of the assembled scaffolds (93.77% of the total) were with gaps of 0–5% (N/length). By clustering and gap-filling, these scaffolds were further assembled into 109,169 unigenes (http://marine.sysu.edu.cn/Public/Uploads/files/4f7ff9d500edc5.rar), including 1622 clusters and 107,547 singletons, with a mean length of 396 bp and an N50 of 478 bp, and 20.71% of which were >500 bp long. 105,206 unigenes (96.37% of the total) contained gaps within 5% in length. Summary statistics of *L. vannamei* transcriptome assembly was shown in Table 2, and the size distributions (>500 bp) of Contigs, Scaffolds, and Unigenes were showed in Fig. 2.

To assess the abundance and coverage of the transcriptome data, we matched the assembled unigenes against the known EST library on Genbank. The 162,926 ESTs downloaded from NCBI were clustered and assembled using TGICL and Phrap, and 25,813 assembled EST-unigenes with mean length of 681 bp and N50 length of 756 bp were generated (http://marine.sysu.edu.cn/Download/download/id/68). Comparisons between transcriptome unigenes and EST-unigenes were performed using BLASTn algorithm. Results were shown in Fig. 3 as a Venn chart and further detailed in Table S1. 60.1% (15,519 out of 25,813) of the EST-unigenes can be matched in the transcriptome unigenes library, whereas only 14.2% (15,519 out of 109,169) of the transcriptome unigene sequences can be found in the ESTs library.

**Figure 4:**
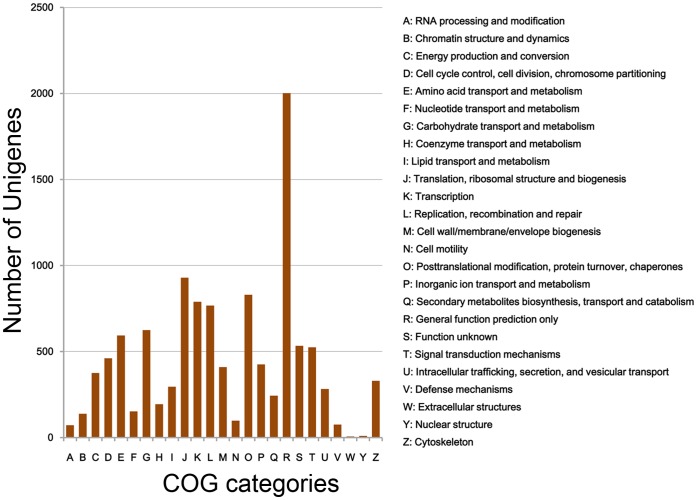
COG Classification of the unigenes. Possible functions of 11153 unigenes were classified and subdivided into 25 COG categories.

### Annotation of Unigenes

After ruling out short-length or low-quality sequences, 73,505 unigenes with a minimum length of 200 bp were selected and subjected to annotation analysis by matching sequences against Nr, Swiss-prot and TrEMBL databases using BLASTx searching with an E value<0.00001. 27,789 unigenes (37.80% of the total) can be matched in Nr database (Table S2), 27,424 (37.3% of the total) matched in Swissprot (Table S3), and 32,439 (44.1% of the total) matched in TrEMBL (Table S4). For main species distribution matched against Nr database, 91.9% of the matched unigenes showed similarities with *Homo sapiens*, followed by *Drosophila melanogaster* (84.2%), *Mus musculus* (72.37%), *Danio rerio* (47.94%), *Rattus norvegicus* (43.17%), *Tribolium castaneum* (43.14%), *Drosophila mojavensis* (38.50%), *Harpegnathos saltator* (37.42%), *Apis mellifera* (37.09%), *Anopheles gambiae str. PEST* (36.62%), *Camponotus floridanus* (35.97%), *Drosophila virilis* (34.95%), *Drosophila willistoni* (33.31%), *Drosophila grimshawi* (32.79%), and *Drosophila yakuba* (32.62%).

The remaining unmatched unigenes (>200 bp) were further analyzed using ESTscan and the predicted CDSs were translated into peptide sequences. CDSs of 11,886 unigenes (16.17% of the total) were successfully predicted by ESTscan.

**Figure 5:**
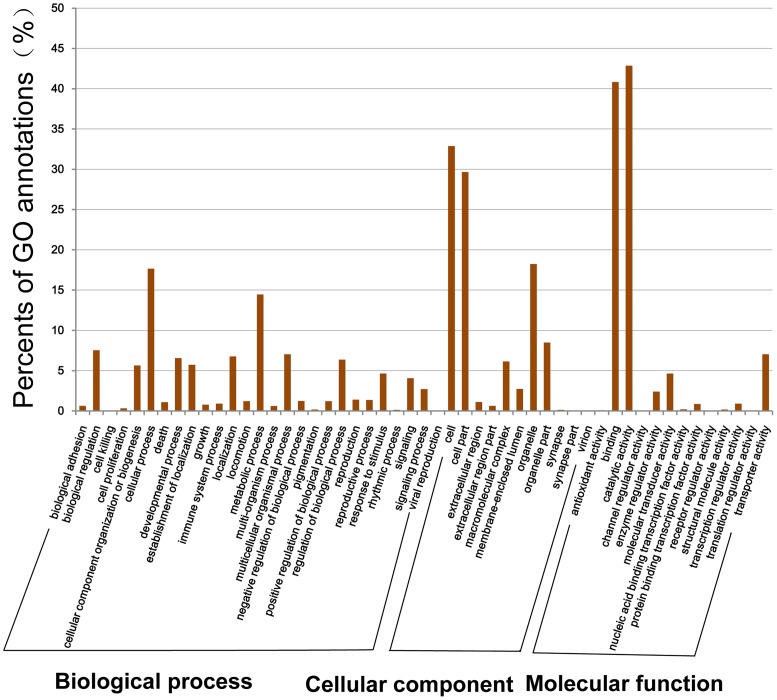
GO categories of the unigenes. 8171 unigenes were assigned 45601 GO annotations, which were divided into three categories: biological processes, cellular components, and molecular functions.

### COG, GO and KEGG Classification

The assembled unigene sequences were subjected to BLAST searching against GO, COG and KEGG databases, and the summary statistics of BLAST assignment was shown in Table 3.

**Figure 6:**
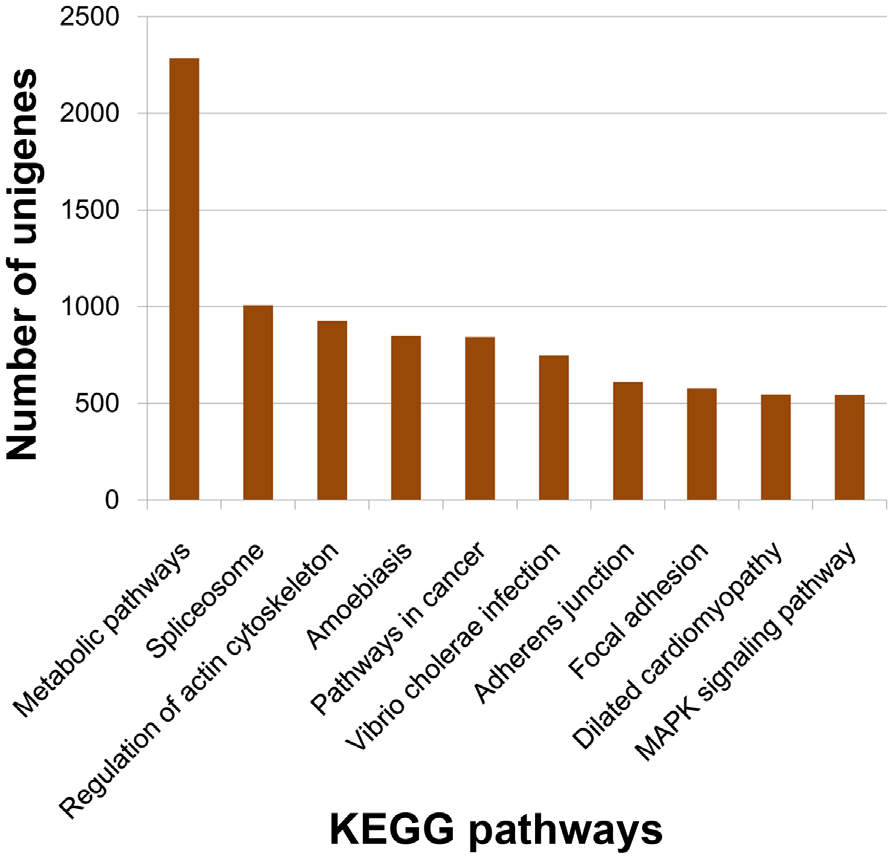
KEGG Classification of the unigenes.18154 unigenes were assigned into 220 KEGG pathways. The top 10 most abundant KEGG pathways are shown.

COG is a database where orthologous gene products were classified. Every protein in COG is assumed to be evolved from an ancestor protein, and the whole database is built on coding proteins with complete genome as well as system evolution relationships of bacteria, algae and eukaryotes [27], [29]. Phylogenetic classifications of the predicted CDSs of unigenes were analyzed by searching against COG database to predict and classify possible functions of the unigenes (Fig. 4). Possible functions of 11,153 unigenes were classified and subdivided into 25 COG categories (Table S5), among which the cluster for ‘General function prediction only’ represents the largest group (2002, 17.95% of the matched unigenes) followed by ‘Translation, ribosomal structure and biogenesis’ (929, 8.33%) and ‘Posttranslational modification, protein turnover, chaperones’ (830, 7.44%). The following categories: ‘extracellular structures’ (6, 0.05%), ‘nuclear structure’ (9, 0.08%) and ‘RNA processing and modification’ (71, 0.64%), represent the smallest groups.

**Figure 7:**
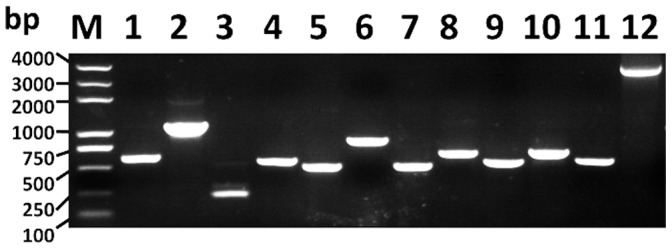
RT-PCR analyses of the amplicons of 12 unigenes related to shrimp embryo development. Amplicons: 1:Unigene98112 (similar to Abdominal-A, 580 bp); 2:Unigene10209 (similar to Abdominal-B, 1036 bp); 3:Unigene54158 (similar to homeotic antennapedia, 216 bp); 4:Unigene16317 (similar to Wnt6, 528 bp); 5:Unigene18019 (similar to Wnt10, 394 bp); 6: Unigene105360 (similar to beta-catenin, 979 bp); 7:Unigene92779 (similar to pumilio, 449 bp); 8:Unigene99210 (similar to pumilio homolog 2, 615 bp); 9: Unigene95206 (similar to Dorsal, 507 bp); 10:Unigene99694 (similar to Spalt, 620 bp); 11:Unigene10400 (similar to gene extra sex combs, 555 bp); 12:Unigene20337 (similar to HIRA, 3941 bp).

GO is an international standardized gene functional classification system which offers a dynamic-updated controlled vocabulary and a strictly defined concept to comprehensively describe properties of genes and their products in any organism [25], [26]. Based on the results of Nr annotation, the Gene Ontology (GO) annotations of unigenes were generated using the BLAST2GO program, and the GO functional classifications were performed using WEGO software to understand the distribution of gene functions of *L. vannamei* from the macro-level (Fig. 5). 45,601 GO term annotations corresponding to 8171 unigenes were produced and assigned into 51 functional groups and three categories, among which 22,268 were assigned in biological process category, 15,403 in cellular component category and 7930 in molecular function category (Table S6). Among the biological process category, ‘cellular process’ (17.65%) and ‘metabolic process’ (14.45%) biological regulation were most highly represented, and other unigenes were categorized into other 25 important biological process, including ‘biological regulation’ (7.53%), ‘multicellular organismal process’ (7.02%), ‘localization’(6.75%), ‘developmental process’ (6.55%), ‘regulation of biological process’ (6.35%), ‘cellular component organization or biogenesis’ (5.64%), and so on. 11 GO functional groups were assigned into the cellular component category, among which ‘cell’ (32.88%) and ‘cell part’ (29.66%) were most highly represented. Similarly, 13 GO functional groups were assigned into the molecular function category, among which ‘catalytic activity’ (42.86%) and ‘binding’ (40.86%) were most highly represented.

**Figure 8:**
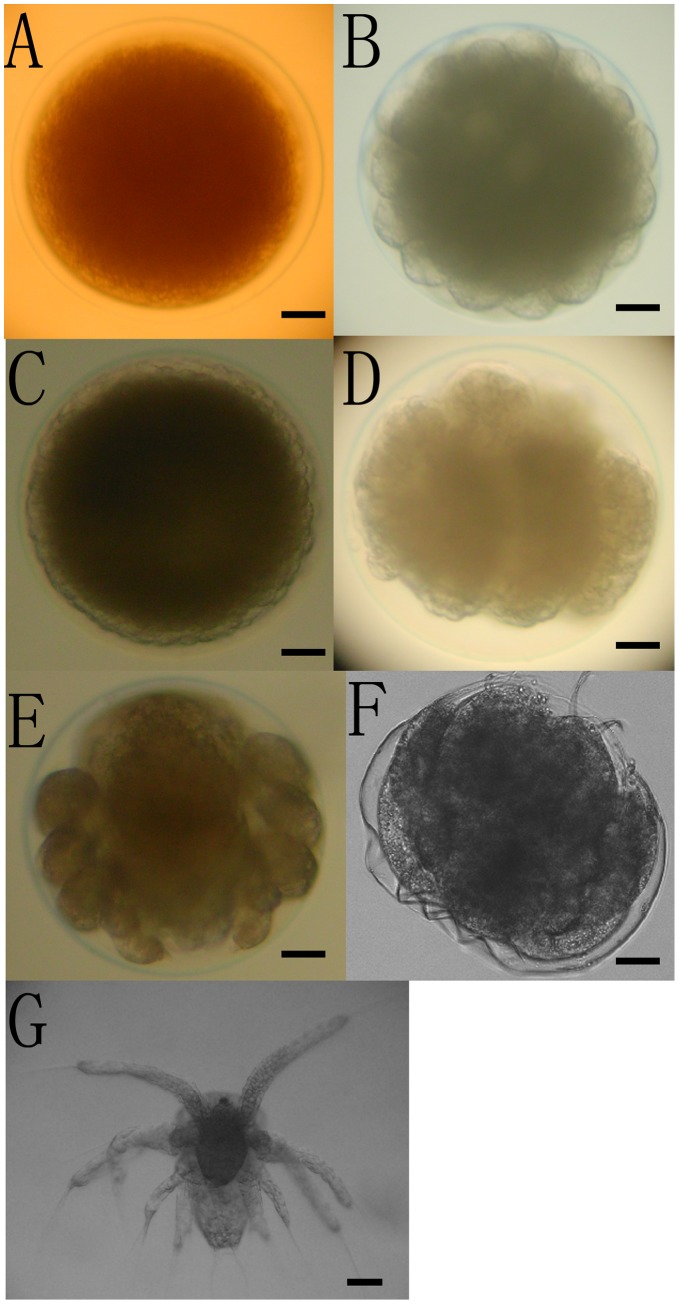
Embryonic development of *L. vannamei* at 28°C, pH 8.0 and 30 g kg ^−**1**^ **salinity.** The sampled *L. vannamei* embryos were checked under the inverted microscope to confirm their embryogenesis stages. A: Newly Spawned Egg (0 mps); B: Blastula Stage (140 mps); C: Gastrula Stage (215 mps); D: Antennal Limb Bud Stage (275 mps); E: Biramous Antenna and Mandible (480 mps); F: Hatching Nauplius (600 mps); G: Newly Hatched Nauplius (660 mps). Abbreviations: mps, minutes post spawning. Scale bars: 100 µm.

**Figure 9:**
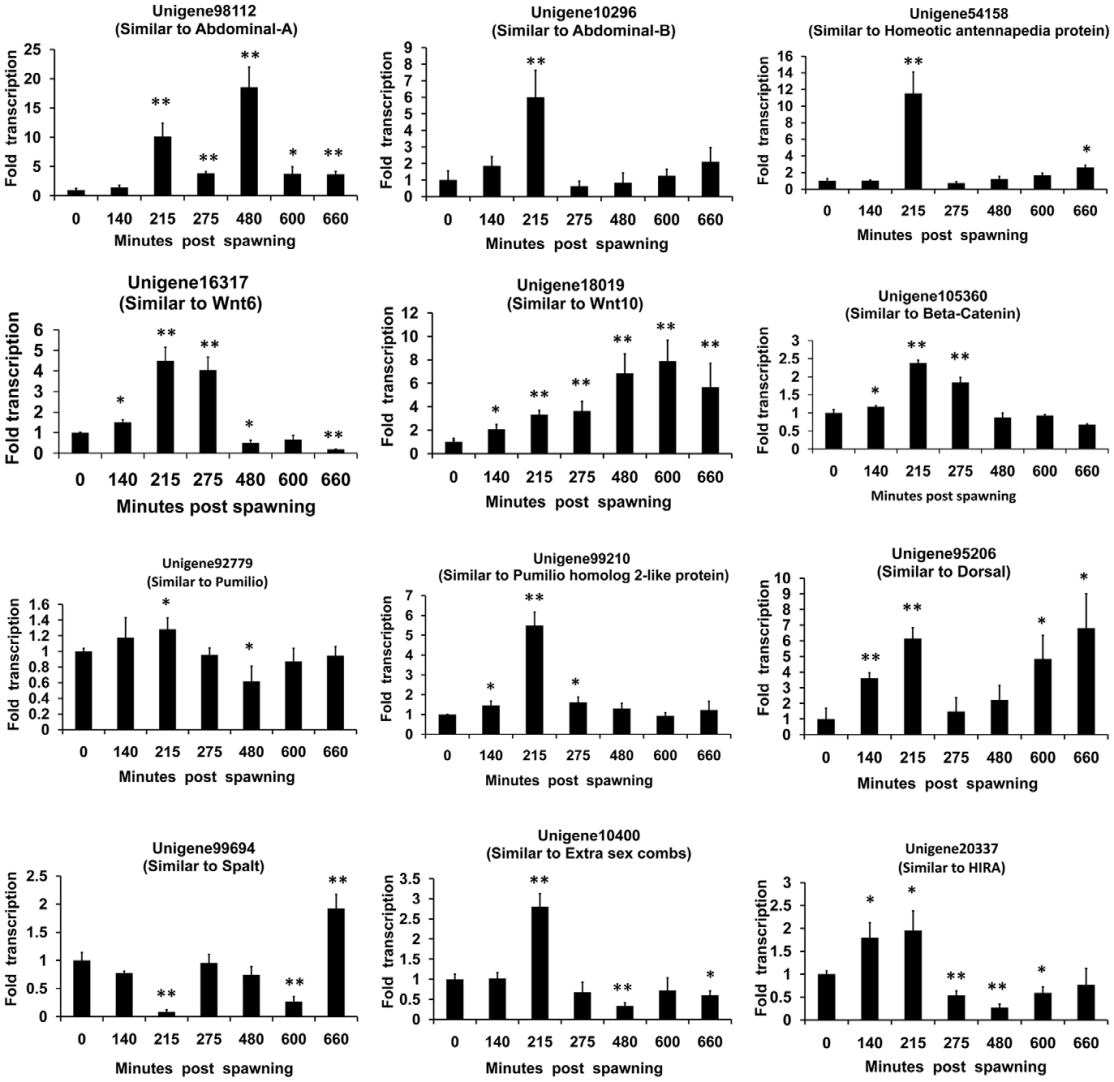
Real-time PCR analyses of the expression profiles of 12 assembled unigenes during embryos development. Experiments were performed in triplicate and repeated three times with similar results. Bars display mean+s.d., and statistical analysis was performed using Student’s T test and the P values were provided (**, P<0.01; *, P<0.05).

The KEGG pathway database records networks of molecular interactions in the cells and variants of them specific to particular organisms. Pathway-based analysis helps us to further learn biological functions of genes [27], [34], [35]. To systematically analyze their inner-cell metabolic pathways and complicated biological behaviors, we classified the unigenes into biological pathways by mapping the annotated CDS sequences to the reference canonical pathways in the KEGG database (Fig. 6). 18,154 unigenes were consequently assigned to 220 KEGG pathways (Table S7), among which 2285 members assigned to ‘metabolic pathways’, followed by ‘Spliceosome’ (1007 members), ‘Regulation of actin cytoskeleton’ (926 members), ‘Amoebiasis’ (848 members), ‘Pathways in cancer’ (843 members), ‘Vibrio cholerae infection’ (748 members), ‘Adherens junction’ (610 members), ‘Focal adhesion’ (576 members), ‘Dilated cardiomyopathy’ (546 members), ‘MAPK signaling pathway’ (545 members), ‘Hypertrophic cardiomyopathy (HCM)’ (539 members), ‘Tight junction’ (507 members), and ‘Pathogenic Escherichia coli infection’ (500 members).

### RT-PCR and Real-time RT-PCR Assays

To primarily verify the results of assemblies and annotations, 12 assembled unigenes that are homologous to many embryo development-related genes, including three Hox family transcriptional regulators abdominal-A, abdominal-B, and homeotic antennapedia (Antp), three Wnt signaling pathway genes Wnt6, Wnt10 and beta-catenin, two Puf Family RNA-binding translational regulators pumilio (PUM) and pumilio homolog 2 (PUM2), a NF-κB family gene Dorsal, a Zn-finger transcription factor Spalt, a Polycomb group (PcG) gene extra sex combs (esc), and histone cell cycle regulation defective homolog A (HIRA), were chosen and subjected to RT-PCR and real-time PCR analyses (Table 1). Full lengths of these unigenes were amplified by RT-PCR using specific primers designed based on the assembly results. The amplicons were analyzed using agarose gel electrophoresis (Fig. 7) and Sanger sequencing (Table S8), which confirmed their lengths and sequences, suggesting the faithful assembly of transcriptome data.

The sampled *L. vannamei* embryos were examined under microscope to determine their embryogenesis stages (Fig. 8). To verify their annotations, expression profiles of the 12 unigenes during embryonic development were further detected using real-time RT-PCR (Fig. 9). The *Strigamia maritima* abdominal-A homolog unigene98122 demonstrated a periodic expression profile with the first peak of 10.14-fold increase at 215 mps and the second peak of 18.54-fold increase at 480 mps, while the *Strigamia maritima* abdominal-B homolog unigene10296 and *Culex quinquefasciatus* Antp homolog unigene54158 showed similar expression profiles with peaks at 215 mps of 6.01-fold and 11.52-fold increase levels, respectively. The Wnt6 homolog unigene16317 peaked at 215 to 275 mps with 4.50–4.04-fold, followed by a sharp decrease at 480 mps, and the Wnt10 homolog unigene18019 kept increasing after spawning and reached a peak at 600 mps with 7.89-flod increase. The unigene105360, similar to beta-catenin, the key molecule of the Wnt pathway, peaked at 215 mps with 2.38-fold and returned to baseline levels after 480 mps. The expression of the *Apis mellifera* PUM homolog unigene92779 was up-regulated during 0–215 mps, and then fell to a low level at 480 mps. The *Saccoglossus kowalevskii* PUM2 homolog unigene99210 exhibited a 5.49-fold increase at 215 mps, and then returned to the basal level at 275 mps where it remained unchanged. The expression of the unigene95206, the dorsal gene of *Litopenaeus vannamei*, was up-regulated periodically, with the first peak of 6.15-fold at 215 mps and the second peak of 6.82-fold at 660 mps. The expression of unigene99694, similar to the spalt protein of *Tribolium castaneum*, was down-regulated during 0–600 mps, with two valleys at 215 mps and 600 mps, and then up-regulated at 660 mps. The unigene10400 (homologous to the esc gene of *Schistocerca Americana*), and unigene20337 (homologous to the HIRA gene of *Takifugu rubripes*) showed similar expression profiles, which up-regulated and peaked at 215 mps and fell to a low value at 480 mps.

## Discussion

Many members of crustacean are of great economic value and important evolutionary status. Up to now, the *Daphnia pulex* genome is the only one sequenced in the subphylum Crustacea, phylum Arthropod [36]. Many transcriptomes of crustaceans have been analyzed using traditional Sanger sequencing and cDNA microarray method, including *Eriocheir sinensis* [37], *Portunus pelagicus* [38], *Petrolisthes cinctipes* [39], *Penaeus monodon* [40], *Penaeus japonicas* [41], *Daphnia magna* [42], *Daphnia pulex* [43], and so on. In recent years, the next generation sequencing methods have also been applied to analyze transcriptomes of crustaceans, such as *Balanus Amphitrite* [44], *Euphausia superb* [45], *Macrobrachium rosenbergii* [46] and *Parhyale hawaiensis* [47] using 454 sequencing, and *Eriocheir sinensis* [48], [49] using Illumina sequencing. Comparing with the traditional methods, the next-generation high-throughput DNA sequencing techniques provide more ideal methods for transcriptome analyses with high efficiency, low cost and high data output. The development of DNA sequencing technology will facilitate the studies on crustaceans’ gene background.

In this study, using the Illumina sequencing method to analyze the trancriptome of *L. vannamei*, more than 2.4 Gb of raw data were generated, and 882,339 contigs (>75 bp) were assembled, largely enriching the transcriptome data of *L. vannamei* and prompting the genome studies of crustaceans. The former studies on *L. vannamei* transcriptome were performed using traditional cDNA library and Sanger sequencing methods with RNA from many organs such as muscle, blood and hepatopancreas. In our study, RNA used for transcriptome analysis was exacted from whole bodies of *L. vannamei* larvae, covering all tissues of the species, which could include fuller transcriptional genes of *L. vannamei*. We compared our transcriptome data with *L. vannamei* EST sequences obtained from NCBI and showed that more than half of the EST sequences (60.1%) can be matched in the transcriptome data, whereas up to 85.8% of the transcriptome unigenes can not be found in the ESTs library. It suggests the transcriptome data provide abundant information besides the now available ESTs sequences.

Although providing much more data throughout than traditional Sanger sequencing method, the reading lengths of the raw data of the Illumina GAII system are quite short. Up to 79.64% of the obtained contigs are less than 100 bp. The SOAP denovo method, a relative mature technique based on the short oligonucleotide analysis package (SOAP) algorithm, was adopted to process the sequencing data, and 73,505 unigenes (>200 bp) with good quality sequences were assembled and subjected to annotation analyses. There are more unigenes showed similarities to *H. sapiens* than other arthropods species such as *T. castaneum, D. mojavensis,* and *H. saltator,* which are phylogenetically closer to *L. vannamei* than human. It is maybe because the now available information on gene background of crustaceans and arthropods is limited, and human genes have been much better studied than other species, providing sufficient gene sequences and annotations for comparison analyses. Only 31.83% of the total analyzed unigenes (84.2% of the Nr database-matched) showed similarities to *D. melanogaster,* a well studied model animal in the Insecta class, phylum Arthropod, maybe because the genome size of *L. vannamei* is almost 12 times more than that of *D. melanogaster* [50] and there might be somewhat different between their gene backgrounds. Further investigation should be required to determine whether protein sequences in crustaceans may have divergence from other animals. With more genes from crustaceans being studied and more gene background information being available, unigenes of *L. vannamei* obtained in this study will be further annotated. Moreover, since it has been reported that there are limitations of the next-generation sequence *de novo* assembly, which could cause missing of the duplicated sequences [51], the possibilities of missing of the encoding sequences in the assembled unigenes might not be excluded. It might also lead to miss-matching of the *L. vannamei* unigenes to protein databases of other species. With the development of sequencing methods and short-read assembly algorithms, further analyses will be performed to rearrange the now available *L. vannamei* transcriptome data and improve their annotations.

Possible functions of the assembled unigenes were analyzed by matching to GO, COG and KEGG databases. Although only a small part of the assembled unigenes were functional annotated, the results of these three databases searching help us learn more about biological features of *L. vannamei*. For example, 748 unigenes can be classified into the ‘*Vibrio cholerae* infection’ KEGG pathway, such as protein disulfide isomerase (PDI, unigene15355), ADP ribosylation factor (ARF, Unigene87975) and transport protein Sec61 (Unigene100471), which have been reported that involve in *Vibrio* infection in arthropods [52]–[55]. It indicated that as an animal living in water, *L. vannamei* may always deal with the challenges from bacteria in water, and may have evolved complicated systems and signal pathways against infection. Interestingly, although no cancer has ever been reported in Arthropoda animals, our results showed that 843 unigenes can be classified into ‘Pathways in cancer’ KEGG pathway. Cancer is a disease of aberrant multicellularity, and its hallmarks are thought to be intimately associated with those of metazoan multicellularity [56], [57]. It has been reported that many ‘multicellularity’ genes of *Amphimedon queenslandica* were also implicated in cancer, suggesting the remote origin of cancer and oncogenes [56], [57]. The KEGG ‘Pathways in cancer’ annotations of these *L. vannamei* unigenes in this study also provided supports for the theme that cancer origin may be related to evolution of multicellularity. Further studies should be performed to confirm this hypothesis.

The 12 unigenes subjected to RT-PCR and real-time PCR analyses were chosen based on the results of annotations, and the primer-pairs were designed based on the sequences of the unigenes assembled with contigs. The products of RT-PCR were sequenced using Sanger sequencing method, which confirmed the sequences of these unigenes, suggesting the faithful results of Illumina sequencing and assemblies. Furthermore, as real-time PCR showed that the expression profiles of these assembled unigenes regularly varied following the developmental stages of embryos, we think the qualities of the annotations of the unigenes may be enough for the requirements of studies on functional genes of *L. vannamei*.

With the development of sequencing techniques, the data of nucleic sequences boosted every day. As more data will be obtained from other species, the assembled unigenes in this study will be further annotated and analyzed. The data of the annotated unigenes are worthy of deeper mining and further analyzing. It will facilitate our understanding of the genome background of crustaceans, and promote the studies on genetics, functional genes, and gene regulations of *L. vannamei.*

## Supporting Information

Table S1 **Comparisons between assembled transcriptome unigenes and the EST-unigenes which were assembled form EST sequences available from Genbank using TGICL and Phrap softwares.**
(ZIP)

Table S2 **BLASTX searching of the Unigenes against NCBI Nr database (E value<0.00001).**
(ZIP)

Table S3 **BLASTX searching of the Unigenes against Swissprot database (E value<0.00001).**
(ZIP)

Table S4 **BLASTX searching of the Unigenes against TrEMBL database (E value<0.00001).**
(ZIP)

Table S5 **COG Classification of the unigenes.**
(ZIP)

Table S6 **GO categories of the unigenes.**
(ZIP)

Table S7 **KEGG Classification of the unigenes.**
(ZIP)

Table S8 **Sanger sequencing of the 12 analyzed unigenes.**
(ZIP)
