# Supplementary material for: Analysis of Litopenaeus vannamei Transcriptome Using the Next-Generation DNA Sequencing Technique
Source: PLoS One. 2012 Oct 11;7(10):e47442. doi: 10.1371/journal.pone.0047442 (PMC3469548; doi:10.1371/journal.pone.0047442)
Supplement: Table S8 — Sanger sequencing of the 12 analyzed unigenes. (ZIP) [file pone.0047442.s008.zip › Table S8.doc]

Table.S8. Sanger sequencing of the 12 analyzed unigenes. The RT-PCR primer sequences were underlined

**1.**

**>Unigene98112_Ex-zai-2 size 586 similar to	abdominal-A [Strigamia maritima]**

GCCGCGTCGTCGTGGACAGCCGTTTGGACTCCATGGCAGGCCGTTCCAATTGGCTGTCAGACCTCGCCATTGGTTTTCTGTGATTGACATCCATGGGTACCGCGGGATGTCCGGGATGTGAGGTGTGGGAGCTGTAGGCTGACTGCAGGAGTTGAGAGGGTTAGTGGCGTTGTTCTGGTGGAAGTACTGTGCCATGTTGCTTGTGGTGGCACAGTTCTGCAGGTTGAGGCCATATTGGGACATGTCCGTGGCCCCAGTCTGGGAGTATCGACACTGCTTTTCGGCGTCTGTGGTCGCCGTCATAGCGCTGAAGGGCGACATGTTGGAGGGCGCGTTGGCAGACAGCTGATGTGAAGTGACACTCACGTAGGGGTACATACTTCTGGCTTGAGAATTGTAGTTGACCAGGTTAGCAGCGGAGTCCGCCTGGTACTTTGGCAGAATACTATCAATATAATTTGAACTCATTGTTCACATAATTCTTCCCAGTGGTAACTACTTAGTAACAACTGTGTACACACCAGCCTCGACTACCTGTGATGGCAAATGAATCCTTTTCTGAATTAGACACCAGGCACGAGAACCG

**Sanger sequencing, size: 580**

TCGTCGTGGACAGCCGTTTGGACTCCATGGCAGGCCGTTCCAATTGGCTGTCAGACCTCGCCATTGGTTTTCTGTGATTGACATCCATGGGTACCGCGGGATGTCCGGGATGTGAGGTGTGGGAGCTGTAGGCTGACTGCAGGAGTTGAGAGGGTTAGTGGCGTTGTTCTGGTGGAAGTACTGTGCCATGTTGCTTGTGGTGGCACAGTTCTGCAGGTTGAGGCCATATTGGGACATGTCCGTGGCCCCAGTCTGGGAGTATCGACACTGCTTTTCGGCGTCTGTGGTCGCCGTCATAGCGCTGAAGGGCGACATGTTGGAGGGCGCGTTGGCAGACAGCTGATGTGAAGTGACACTCACGTAGGGGTACATACTTCTGGCTTGAGAATTGTAGTTGACCAGGTTAGCAGCGGAGTCCGCCTGGTACTTTGGCAGAATACTATCAATATAATTTGAACTCATTGTTCACATAATTCTTCCCAGTGGTAACTACTTAGTAACAACTGTGTACACACCAGCCTCGACTACCTGTGATGGCAAATGAATCCTTTTCTGAATTAGACACCAGGCACGAGAACCG

**2.**

**>Unigene10296_Ex-zai-2 size 1049 similar to	abdominal-B [Strigamia maritima]**

GGCTAGGGGCAGAGGGGTGACAGGAACTCTGCACTTGTCAAGGCTTGATGCTGTTTGGAGTCTGGGGGGTGTTGGGCTGGTGGCCGGGGGTGCCGCCCCCGGAGGAAGGAGTGCCAGCCGCGGCAGCCGCCGCCGCCTCCCCTGTCTTTGGCTATTTTTCTTGTTTTTCATTCGACGATTCTGGAACCATATTTTAACCTGTCGCTCCGTGAGGTTGAGGTTGCGGGCGAGCTCCCAGCGCTTCTGCTTGGACACGTAGGCGTTGTACAGGAACTCCTTCTCCAACTCCAGCGTCTGGAACTTGGAGTAGGGCTNNNNNNNNNNNNNNNNNNNNNNNNNNNNNNNNNNNNNNCCGTCCACTCCAGCGGGTTGGAGCCCAGCGCGCCTGGATGTGAGACGGGGAAGGGCGGCCGGTGCGCTGGAGGGTAAGGTGCGGGGGGCATAGTCTCTGCTGTGGGGTAGTTCCTCAGGTAGCCGTCGTGGGGATATCCTGCGGCAGCTTCCATGGCAGATTTTGGGGCACCATCTGCCGCCGNNNNNNNNNNNNNNNNNNNNNNCCGTGGGCATCGACATGGCCCACACGCCCGTAGGGAGGGTAGTTGCACCAGGTCTGAGCGGCAAAGGCTTGGCATGACTCCGTGGTCATCGAGGATGGCCCCGGTGCGTAGTCGTATTTGTTGGGCCAGAAGGCCAGAGTCTTGCGGTCAGCCACTCTGGTGTCCGCAATGTTATAGTACGTGGGCGTGACCCCGTCCCGCCCGAAGGCCTGGCCGTACTGGGAGTCGAAGGGCGCCGTGGCTGCTGCATGCTCCGGTTGGTAGTTCCAGGGCTGTGTGGAGTGCGAGTGCCTGATGACACCCGCCGCGGTGCCAGAGTCAGGCCCCGTGGGGCACTCGGGCGGGTGCGGGTACGTGGACACAGCCAGGGTGGTGGCCACGCGCTTGGCCGGGATGTGTATTGGTGTTTGGCCCGTAGGCTCCAGGCCTCGTGTGCCGGCCGAGTCCTCATACAAAGCTCCGTTCATCCTGTCTAGTCCTCCTCCCTCGCTG

**Sanger sequencing, size: 1036**

GGCTAGGGGCAGAGGGGTGACAGGAACTCTGCACTTGTCAAGGCTTGATGCTGTTTGGAGTCTGGGGGGTGTTGGGCTGGTGGCCGGGGGTGCCGCCCCCGGAGGAAGGAGTGCCAGCCGCGGCAGCCGCCGCCGCCTCCCCTGTCTTTGGCTATTTTTCTTGTTTTTCATTCGACGATTCTGGAACCATATTTTAACCTGTCGCTCCGTGAGGTTGAGGTTGCGGGCGAGCTCCCAGCGCTTCTGCTTGGACACGTAGGCGTTGTACAGGAACTCCTTCTCCAACTCCAGCGTCTGGAACTTGGAGTAGGGCTTCCGCTTCTTCCGGACCGTCATGTTCCCCGTCCACTCCAGCGGGTTGGAGCCCAGCGCGCCTGGATGTGAGACGGGGAAGGGCGGCCGGTGCGCTGGAGGGTAAGGTGCGGGGGGCATAGTCTCTGCTGTGGGGTAGTTCCTCAGGTAGCCGTCGTGGGGATATCCTGCGGCAGCTTCCATGGCAGATTTTGGGGCACCATCTGCCGCCGTTAGGTATGGCACGGGCTGGCCGTGGGCATCGACATGGCCCACACGCCCGTAGGGAGGGTAGTTGCACCAGGTCTGAGCGGCAAAGGCTTGGCATGACTCCGTGGTCATCGAGGATGGCCCCGGTGCGTAGTCGTATTTGTTGGGCCAGAAGGCCAGAGTCTTGCGGTCAGCCACTCTGGTGTCCGCAATGTTATAGTACGTGGGCGTGACCCCGTCCCGCCCGAAGGCCTGGCCGTACTGGGAGTCGAAGGGCGCCGTGGCTGCTGCATGCTCCGGTTGGTAGTTCCAGGGCTGTGTGGAGTGCGAGTGCCTGATGACACCCGCCGCGGTGCCAGAGTCAGGCCCCGTGGGGCACTCGGGCGGGTGCGGGTACGTGGACACAGCCAGGGTGGTGGCCACGCGCTTGGCCGGGATGTGTATTGGTGTTTGGCCCGTAGGCTCCAGGCCTCGTGTGCCGGCCGAGTCCTCATACAAAGCTCCGTTCATCCTGTCTAGTCCTCCTCCCTCGCTG

**3.**

**>Unigene54158_Ex-zai-2 size 216 homeotic antennapedia protein [Culex quinquefasciatus]**

CAGCTCGAGGGTCTGGTACCGCGTGTAGGACGTTCTCTGGCGCTTCGTCTCGCCGTTGGAGTTCACTGTACCTTTTCCGCCATCCTTCTGATTCTGGCCGAGGTGCACTCTCTTCATCCACGGGTAGATCTGAGGGGCGGGCTTCCCGGAGGACGAGCCTCCCGCGCTTCCAGAGGCGCCCTCCAGACTCTCTCCTCCTTCGGCTCCTTCGCCTCC

**Sanger sequencing, size:216**

CAGCTCGAGGGTCTGGTACCGCGTGTAGGACGTTCTCTGGCGCTTCGTCTCGCCGTTGGAGTTCACTGTACCTTTTCCGCCATCCTTCTGATTCTGGCCGAGGTGCACTCTCTTCATCCACGGGTAGATCTGAGGGGCGGGCTTCCCGGAGGACGAGCCTCCCGCGCTTCCAGAGGCGCCCTCCAGACTCTCTCCTCCTTCGGCTCCTTCGCCTCC

**4.**

**>Unigene16317_Ex-zai-2 size 555 gap 0 0% similar to WNT6 [Monodelphis domestica]**

CGGCAGCCAGGACATCCGCTCCATCGTGATGCTCCACAACAACGAGGTCGGGCGGCTGGCTGTGCAGCGGCACCTTCAGCCCCATTGCAAGTGCCACGGCCTCTCCGGCTCCTGCACTCACCGCACCTGCTGGAAGAGACTGCCCACCTTCAGGTCCATCGGCGCGAGACTCAAGGAAAAATTCAGACAGGCTATTAAGGTGACTCCGAGCAACGACGGAACGAGTATCCGTCCAGTGGGCCAGTTAACAGTCAGTGACGAGGACCTCATCTTCCTGGAGGATTCGCCGGACTTCTGCAGGCCTAACAAGCGAACGGGGTCACTGGGTACACAAGGTCGGATGTGCAACGTAACAGCGAATGACATGTCTGGCTGTGACCTGATGTGCTGCGGGCGTGGAATCAGACAGGAGGTATTGGAGCTGGAAGAGAACTGCAGATGTCGCTTCAAGTTCTGCTGTGAAGTCACCTGCCAGAAGTGTCGAATCAAGAGGAAGATGTCTTACTGCCTCTGAGCTCAGCATGATTACGGAATAATTGATGATAGCGTCCGGGG

**Sanger sequencing, size: 528**

CCATCGTGATGCTCCACAACAACGAGGTCGGGCGGCTGGCTGTGCAGCGGCACCTTCAGCCCCATTGCAAGTGCCACGGCCTCTCCGGCTCCTGCACTCACCGCACCTGCTGGAAGAGACTGCCCACCTTCAGGTCCATCGGCGCGAGACTCAAGGAAAAATTCAGACAGGCTATTAAGGTGACTCCGAGCAACGACGGAACGAGTATCCGTCCAGTGGGCCAGTTAACAGTCAGTGACGAGGACCTCATCTTCCTGGAGGATTCGCCGGACTTCTGCAGGCCTAACAAGCGAACGGGGTCACTGGGTACACAAGGTCGGATGTGCAACGTAACAGCGAATGACATGTCTGGCTGTGACCTGATGTGCTGCGGGCGTGGAATCAGACAGGAGGTATTGGAGCTGGAAGAGAACTGCAGATGTCGCTTCAAGTTCTGCTGTGAAGTCACCTGCCAGAAGTGTCGAATCAAGAGGAAGATGTCTTACTGCCTCTGAGCTCAGCATGATTACGGAATAATTGATGATAGCG

**5.**

**>Unigene18019_Ex-zai-2 size 408 similar to Wnt10 CG4971-PA [Tribolium castaneum] NCBI: Aedes aegypti Wnt10a**

CTGGCACCGAACCGGGTGTTGTCGTGGCACCCGGACCACTTCCACGCTCTCTTGCCTCGAACCATGCCGCAGGAGCAGTTGTGGAGGCGCCCAGCTGCGCATGCGCGGGCGACACTGTGGGTGACTCCAGCTGCCGTGATGGCGTGACCGAAGGCGCTCTCGCTGTAGCCTCTTGCCATGATAGCAGCTGTGTGCGGGTTGTTGGTCGGTGTGGTAAGGGCAGTGCAGTTCCAGCGGTGATTCGCAAACTGCCTTCCGCACTCTCTAACTGCTAGTTGGAGGCCAATAGCTGCTGCTCCTGCTAGTTCGACGTTGCGCGTGCAGACGTGGAGCTGCGACCTCGTCAGCCCCGACATGGTCCTGCAGACGTCGGGGTCGAGCACGTGGTCCCAGACCGCGGCGTCCCAC

**Sanger sequencing, size: 394**

CTGGCACCGAACCGGGTGTTGTCGTGGCACCCGGACCACTTCCACGCTCTCTTGCCTCGAACCATGCCGCAGGAGCAGTTGTGGAGGCGCCCAGCTGCGCATGCGCGGGCGACACTGTGGGTGACTCCAGCTGCCGTGATGGCGTGACCGAAGGCGCTCTCGCTGTAGCCTCTTGCCATGATAGCAGCTGTGTGCGGGTTGTTGGTCGGTGTGGTAAGGGCAGTGCAGTTCCAGCGGTGATTCGCAAACTGCCTTCCGCACTCTCTAACTGCTAGTTGGAGGCCAATAGCTGCTGCTCCTGCTAGTTCGACGTTGCGCGTGCAGACGTGGAGCTGCGACCTCGTCAGCCCCGACATGGTCCTGCAGACGTCGGGGTCGAGCACGTGGTCCCAGA

**6.**

**>Unigene105360_Ex-zai-2 size 979 similar to beta-catenin [Parhyale hawaiensis]**

GACTTTGGTTGCAGCATCTGAGAGGTTACGCAGCGTCCACAGGCAGTTTTGGACAAGGCGGGTTGACTGGTGTGTTAGGTGCATGGCAAGTGCCTGCATGCCACCAGCTTCAACAATGGCTGGCTTGTTACTTGAACACACAGACAAAACTTTGAGCACCCTGGAGGTCGTCCACAAGAGCTTCTCATAGGTGTAGGAGCGTAGGATTCGAACCAGCTCCTGAGGTCCCTGAGATGCTAAGATAATCAGTTTGGATTCCTGGTTGCCATACGCCAAGATCTGCAAGCAGTCTGTGACGATGGCAAGGAACTTGACATTGTTGCGTTGGAGCAGGGCGACCATCTTCTGGAGACCTCCGGCCATGCGCACTGCAGTCTTAGAGCCTTCCTGGTGCAGCAGGAGGTTGTGGAGTGTTGTGATGGCATAGAACAACACTGACTCTACAGGTGAGCTAAGAAGTTTCACCAATGCTGGAATGCCACCAGACTTGAAAATGGCTAATAAACCCTGGCGATGATGAGACAAATTATGCAGGGCCCCAACAGTGACACGGGTTGTCTCCAGGTCATTGGAATTGGTGAGGGCACGCACGAGGGCAGCCACCATCTGTTGGGAGTTCATGATGGCATGGCGTGAGGCCTCCTTCTTGCTGAGGCGATGGACCATCATGGCAGCCTGGGAGACCACCACCTGATCCTCATCATTGAGCAGTTTGATCAACTCTGGGATGGCGCGAGTTGCCAGATCAGCATCATCCTGATAGTTGATTAAGTTGACAACAGCATGTTTGAGCATTTGTGAGGGTTCCGCCAACCTCTGCACAGCTGTAGGCTGCTGGGGGTCAAACTGGGTGGAGGGGATCTCCATGCCCTCATCCAGTGTCTCTGGGAACATGGCAGCTCGGACACGCTGGGAACGTGTCTGATTCAGCTGCTGGTTCATGTCATCGACCTGCTCGCTGGTGAAGGCAGCCTGGTTG

**Sanger sequencing, size: 979**

GACTTTGGTTGCAGCATCTGAGAGGTTACGCAGCGTCCACAGGCAGTTTTGGACAAGGCGGGTTGACTGGTGTGTTAGGTGCATGGCAAGTGCCTGCATGCCACCAGCTTCAACAATGGCTGGCTTGTTACTTGAACACACAGACAAAACTTTGAGCACCCTGGAGGTCGTCCACAAGAGCTTCTCATAGGTGTAGGAGCGTAGGATTCGAACCAGCTCCTGAGGTCCCTGAGATGCTAAGATAATCAGTTTGGATTCCTGGTTGCCATACGCCAAGATCTGCAAGCAGTCTGTGACGATGGCAAGGAACTTGACATTGTTGCGTTGGAGCAGGGCGACCATCTTCTGGAGACCTCCGGCCATGCGCACTGCAGTCTTAGAGCCTTCCTGGTGCAGCAGGAGGTTGTGGAGTGTTGTGATGGCATAGAACAACACTGACTCTACAGGTGAGCTAAGAAGTTTCACCAATGCTGGAATGCCACCAGACTTGAAAATGGCTAATAAACCCTGGCGATGATGAGACAAATTATGCAGGGCCCCAACAGTGACACGGGTTGTCTCCAGGTCATTGGAATTGGTGAGGGCACGCACGAGGGCAGCCACCATCTGTTGGGAGTTCATGATGGCATGGCGTGAGGCCTCCTTCTTGCTGAGGCGATGGACCATCATGGCAGCCTGGGAGACCACCACCTGATCCTCATCATTGAGCAGTTTGATCAACTCTGGGATGGCGCGAGTTGCCAGATCAGCATCATCCTGATAGTTGATTAAGTTGACAACAGCATGTTTGAGCATTTGTGAGGGTTCCGCCAACCTCTGCACAGCTGTAGGCTGCTGGGGGTCAAACTGGGTGGAGGGGATCTCCATGCCCTCATCCAGTGTCTCTGGGAACATGGCAGCTCGGACACGCTGGGAACGTGTCTGATTCAGCTGCTGGTTCATGTCATCGACCTGCTCGCTGGTGAAGGCAGCCTGGTTG

**7.**

**>Unigene92779_Ex-zai-2 size 468 PREDICTED: similar to pumilio [Nasonia vitripennis]**

TTAAAAGTTTTGTCGTCATCGAGGCCGTTCTGCACGATGCCGTTCGCCTGTGGGGCGTTGCCGTTCTCTACGTCCTTCTTTGTGGGGTCGAAAGGTGACGGTGCCTTATCTTTCTTGTCCTTGCTCGTCTCTACACCACCGTTTCTGAGTTGCAGGTTGGCCATGCGAGCGTCTAGGTCCTTGCCTGTGGGTGAGGACGCCAACACGTACTCGACCATCTTGAGCCCCACACCGGCGTGGTCCGAAGCGCGCGGGGACAGCACGGAGTCTCCCCCGGGGAACTGTGGACGTCCGGGGCCTCGCTGCCCCACACCCATACTCAACCCGTGGCTCTGTCCCACTCCGTGGTCTGTTGGGTGTCCTGTGCTCCATGCTGCTTCTCTCCAAGTGGGTTCACCTATGTTGAAAATCCCCTTGTTGTCTGTTTTCCCCTCTTCCACTCCCCACAGCTTTTTCGCTGGCGCAATC

**Sanger sequencing, size: 449**

GTTTTGTCGTCATCGAGGCCGTTCTGCACGATGCCGTTCGCCTGTGGGGCGTTGCCGTTCTCTACGTCCTTCTTTGTGGGGTCGAAAGGTGACGGTGCCTTATCTTTCTTGTCCTTGCTCGTCTCTACACCACCGTTTCTGAGTTGCAGGTTGGCCATGCGAGCGTCTAGGTCCTTGCCTGTGGGTGAGGACGCCAACACGTACTCGACCATCTTGAGCCCCACACCGGCGTGGTCCGAAGCGCGCGGGGACAGCACGGAGTCTCCCCCGGGGAACTGTGGACGTCCGGGGCCTCGCTGCCCCACACCCATACTCAACCCGTGGCTCTGTCCCACTCCGTGGTCTGTTGGGTGTCCTGTGCTCCATGCTGCTTCTCTCCAAGTGGGTTCACCTATGTTGAAAATCCCCTTGTTGTCTGTTTTCCCCTCTTCCACTCCCCACAGCTTTTT

**8.**

**>Unigene99210_Ex-zai-2 size 619 PREDICTED: pumilio homolog 2-like [Saccoglossus kowalevskii]**

CTTCTGGCGGATCCTCATCCTTATTCCCACCCATAAAACGAAACCTGAGCTTGGAAAAATGTACTGGTCGCTCACGCTTACTGGAGGACTTCCGAAACAATCGATTCCCCAACCTGCAGCTCAGAGACTTGGCCAACCACATAGTGGAATTCAGCCAAGACCAGCATGGTTCCAGGTTCATTCAACAAAAGCTTGAACGAGCAACACCAGCTGAAAAGCAGATGGTATTCAACGAAATCCTAACAGCAGCTTATTCTTTAATGACTGATGTGTTTGGTAATTACGTGATTCAGAAGTTCTTTGAATTTGGAACCCCCGAGCAGAAGACTGTCTTGGCAAACAAGATTCGTGGCCACGTTTTGCCTTTAGCACTACAGATGTATGGATGCCGTGTGATTCAGAAGGCTCTTGAGTGCATTACTCAGGACCAGCAGAAAGACATCGTGCGTGAACTGGATGGCCATGTCCTCAAATGTGTTAAGGATCAGAATGGCAACCATGTCGTGCAAAAATGCATTGAGTGTGTGGATCCCATGGCCTTACAGTTTATCATCAATGCTTTCCAGGGGCAAGTCTTCACCCTCTCTACACACCCCTATGGTTGCCGTGTTATTCAGCG

**Sanger sequencing, size: 615**

CTGGCGGATCCTCATCCTTATTCCCACCCATAAAACGAAACCTGAGCTTGGAAAAATGTACTGGTCGCTCACGCTTACTGGAGGACTTCCGAAACAATCGATTCCCCAACCTGCAGCTCAGAGACTTGGCCAACCACATAGTGGAATTCAGCCAAGACCAGCATGGTTCCAGGTTCATTCAACAAAAGCTTGAACGAGCAACACCAGCTGAAAAGCAGATGGTATTCAACGAAATCCTAACAGCAGCTTATTCTTTAATGACTGATGTGTTTGGTAATTACGTGATTCAGAAGTTCTTTGAATTTGGAACCCCCGAGCAGAAGACTGTCTTGGCAAACAAGATTCGTGGCCACGTTTTGCCTTTAGCACTACAGATGTATGGATGCCGTGTGATTCAGAAGGCTCTTGAGTGCATTACTCAGGACCAGCAGAAAGACATCGTGCGTGAACTGGATGGCCATGTCCTCAAATGTGTTAAGGATCAGAATGGCAACCATGTCGTGCAAAAATGCATTGAGTGTGTGGATCCCATGGCCTTACAGTTTATCATCAATGCTTTCCAGGGGCAAGTCTTCACCCTCTCTACACACCCCTATGGTTGCCGTGTTATTCAGC

**9.**

**>Unigene95206_Ex-zai-2 size 514 dorsal [Litopenaeus vannamei]**

CTCAAGTTACGAGAGGAGATTAGAGTGGACCCTTTCCAGACCGGGTTTTCTCATCGTAACCAGCCACAGAGTATTGATCTTAATGCTCTGAGATTGTGTTTTCAAGTATTCCTCGAGGGATCAGAAAAGGGGAAATTTACTTTCCCGTTAAAACCACAGGTTTCAGATCCAATTTATGACAAAAAGGCCACATCAGATCTGATTATCTGCAAGCTCAGTGACTGCACAAGCAGTGTGGCTGGGGGCAAAGAGATTATTCTCCTCTGTGATAAAGTAACTAAAGAGGACATTCATGTCCGTTTCTATGAGGTCAAAGATGGAATGATAGAATGGGAAGCCTTCGGTGATTTCCAAGCTTCTGATGTCCACAAGCAAGTGGCCATTTCGTTTAAAACTCCCAGATATAAAACTCTTGAGATAGAGAACCCCATAAAGGTGTACGTGCAGCTGTTGCGACCATCAGACAAGAGTACCAGTGAGCCACGGCCATTCCAGTATTTGCCTCTAGACTCAG

**Sanger sequencing, size: 507**

AGTTACGAGAGGAGATTAGAGTGGACCCTTTCCAGACCGGGTTTTCTCATCGTAACCAGCCACAGAGTATTGATCTTAATGCTCTGAGATTGTGTTTTCAAGTATTCCTCGAGGGATCAGAAAAGGGGAAATTTACTTTCCCGTTAAAACCACAGGTTTCAGATCCAATTTATGACAAAAAGGCCACATCAGATCTGATTATCTGCAAGCTCAGTGACTGCACAAGCAGTGTGGCTGGGGGCAAAGAGATTATTCTCCTCTGTGATAAAGTAACTAAAGAGGACATTCATGTCCGTTTCTATGAGGTCAAAGATGGAATGATAGAATGGGAAGCCTTCGGTGATTTCCAAGCTTCTGATGTCCACAAGCAAGTGGCCATTTCGTTTAAAACTCCCAGATATAAAACTCTTGAGATAGAGAACCCCATAAAGGTGTACGTGCAGCTGTTGCGACCATCAGACAAGAGTACCAGTGAGCCACGGCCATTCCAGTATTTGCCTCTAGACT

**10.**

**>Unigene99694_Ex-zai-2 size 635 similar to spalt [Tribolium castaneum]**

TCAGGTACCAGCTGTCCCCCGAGGCCCTCAGGCACCGGGAGATGATGGAGAGCGAAGACCGGGGCAACGAGGAAGAGAAGTACCTTCGTCCCTTCAGCAACTCCTCCAGGTCGTCCTCGACGGGCAGCGCCGAGCAGCGCACCGCCGAGGACCTGACGGTGAGGGCTCGCGAGGACAACTACAACGGCATGACCTCCCCCCGCGCCTCCGTCTCGCCCACGCCGTCGGACTACTCCGACCTAGGGGAGAGGTCGCATGACGCTCCCGCTGACAGTCAGGTGTCGCCGCCCGACCACAATATCAGCGGCTCCGTCAGCCCCGCCGTCAGCACAGGAAGTCAGGACGGAGGAGTGCCTCTGCCCCTGGACTTGGCGCAGCGCTCTCACCCGCACCTGTTCTCGCCCTTCGGCCTGTTCCCGCCGCCCCTCACGACGGGCCCGCTGACGCCCGGCACCCACTCCATGCCGGGCCTCAACCCGCTCTACATGCCCAGCATGCCAGGCCGCGGCAACACCACGTGTCAGATTTGCTTCAAAACCTTCGCTTGCCAATCGGCGCTTGAGATCCACATCCGATCCCACACGAAGGAGCGGCCCTTCAAGTGCAGCATCTGCGACCGAGGCTTCTCAACTAAG

**Sanger sequencing, size: 620**

GCTGTCCCCCGAGGCCCTCAGGCACCGGGAGATGATGGAGAGCGAAGACCGGGGCAACGAGGAAGAGAAGTACCTTCGTCCCTTCAGCAACTCCTCCAGGTCGTCCTCGACGGGCAGCGCCGAGCAGCGCACCGCCGAGGACCTGACGGTGAGGGCTCGCGAGGACAACTACAACGGCATGACCTCCCCCCGCGCCTCCGTCTCGCCCACGCCGTCGGACTACTCCGACCTAGGGGAGAGGTCGCATGACGCTCCCGCTGACAGTCAGGTGTCGCCGCCCGACCACAATATCAGCGGCTCCGTCAGCCCCGCCGTCAGCACAGGAAGTCAGGACGGAGGAGTGCCTCTGCCCCTGGACTTGGCGCAGCGCTCTCACCCGCACCTGTTCTCGCCCTTCGGCCTGTTCCCGCCGCCCCTCACGACGGGCCCGCTGACGCCCGGCACCCACTCCATGCCGGGCCTCAACCCGCTCTACATGCCCAGCATGCCAGGCCGCGGCAACACCACGTGTCAGATTTGCTTCAAAACCTTCGCTTGCCAATCGGCGCTTGAGATCCACATCCGATCCCACACGAAGGAGCGGCCCTTCAAGTGCAGCATCTGCGACCGAGGCTTCTCAA

**11.**

**>Unigene10400_Ex-zai-2 size 567 extra sex combs [Schistocerca americana]**

CAAGCATTTTGTTGGTCATGGAAATGCTATCAATGAACTCAAGTTCCATCCTCGGGACCCAAACCTCCTCCTGTCTGTAAGCAAGGACCATGCACTGAGGATGTGGAACATACGTACGGATATTCTTGTTGCCCTCTTTGGAGGTGTGGAAGCGCACAGGGATGAAGTGCTGAGTGCAGATATAGATGTGGAAGGAACTTGCATAGTATCCTGTGGAATGGATCACTCGCTGAAGATTTGGAAATTAACCTCGGACATAATGCTCAAGACCATCAATCACTCGTACACTTTCAACCCAAGCAGAGCAGTGCAACCCTTTCCAACACTCCAGCAAAATTTTCCCGACTTCTCCACGAGGGACATCCATAGAAACTATGTGGATTGTGTGCGTTGGTTAGGAAAATTTATCCTCTCTAAGAGCTGTGAAAATACAATAGTGTGTTGGAAGCCAGGCTTACTGAACCAGACTGAATTGAAGCACAATGATACCAATGTCACAATTATTCAGAAATTTGATTACAAGGAATGTGAGATCTGGTTTATGAGGTTTGCTTTGGACTTTTGGCA

**Sanger sequencing, size: 555**

GCATTTTGTTGGTCATGGAAATGCTATCAATGAACTCAAGTTCCATCCTCGGGACCCAAACCTCCTCCTGTCTGTAAGCAAGGACCATGCACTGAGGATGTGGAACATACGTACGGATATTCTTGTTGCCCTCTTTGGAGGTGTGGAAGCGCACAGGGATGAAGTGCTGAGTGCAGATATAGATGTGGAAGGAACTTGCATAGTATCCTGTGGAATGGATCACTCGCTGAAGATTTGGAAATTAACCTCGGACATAATGCTCAAGACCATCAATCACTCGTACACTTTCAACCCAAGCAGAGCAGTGCAACCCTTTCCAACACTCCAGCAAAATTTTCCCGACTTCTCCACGAGGGACATCCATAGAAACTATGTGGATTGTGTGCGTTGGTTAGGAAAATTTATCCTCTCTAAGAGCTGTGAAAATACAATAGTGTGTTGGAAGCCAGGCTTACTGAACCAGACTGAATTGAAGCACAATGATACCAATGTCACAATTATTCAGAAATTTGATTACAAGGAATGTGAGATCTGGTTTATGAGGTTTGCTTTGGA

**12.**

**>Unigene20337_Ex-zai-2 size 4036 protein HIRA [Takifugu rubripes]**

CAAAGATGGCGGAGGAAGCGTCTTAGCGATTTAGGCGGCGTTTGAATAATGTCTTTGTGTGATTGGAAAGAGACAGAACAAGTATCTTGTTTGTTCTGAAGAGGCTGGAAGTACCATGAAGCTCCTAAAACCGCCTTGGATCAACCATGATGGTGACAAGGCAATCTTCAGTGTTGACCTACATCCTGATGGTTCAAGATTTGCGACAGGTGGACAAGGACAGGATTCCTCTGGAAGGGTTGTGGTGTGGAACCTGAAACCAGTTGCTTTAGAGAAATATGAGGAAGATGAGAAGGTACCGAAGTTGTTGTGTATGCTAGACCAGCACTTGGGGTGTGTGAATTGTGTGAGGTGGTCTCAGAGTGGACGTTTCTTAGCTTCTGCTGGAGACGATAAAGTTATTATCATCTGGCAGCAGAGCAGTTATGGAGGTGGAGCTGTATTTGGTTCAAATGTTGTGAATGTTGAATCTTGGCGAAGTGTCACAACACTAAGAGGCCACAATGGGGATATCCTCGATGTAGCTTGGTCTCCAGGGGATGTTTGGCTAGCATCTGCTTCTGTAGACAATAATGTTATTGTATGGAATACTGCAAAATGGCCAGAACAAGTGCAGATTCTTCGTGGACATACAGGATTGGTGAAAGGTGTTACTTGGGATCCTGTTGGTCGTTACTTAGCCTCCCAGGCAGATGACAAAAGTCTAAGGATTTGGCGAACAACTGACTGGACCACAGAAGAAAAAATAATTGACCCTTTCAGTGAGTGTGGTGGCACAACAACAGTCCTTCGGCCAAGCTGGTCACCTGATGGTCAGTATCTGGTCTCTGCACATGCAATGAACAATGGAGGTCCTACTGCACAGATTGTAGAAAGAGATGGATGGAACACTGACAAGGATTTTGTTGGCCATCGTAAACCTATCACGTGTGTGAGGTTTAATCCTAATATCCTGGAGAAAGCTGATCCAAAGACAGGGAAGTCTGTGCAGTACTGTTGTGTGGCAATTGGCTCCAGGGATCGCTCAATCTCTATTTGGTTTACATCGCTGAAGAGACCTTTGGTGGTGATTCATGATATATTTGATGACTCAGTTCTAGATCTTTCATGGAGCAGTTGTGGCAAGTATCTCATGGCAGTGTCAAAAGATGGTTCTTTGGCCTTCATAAACTTTTCCCCAGATGAGATTGGCCGTCCACTTTGTGAAGATGAAACTAATAACCTCCACATGAAGTTGTACGGTAAATCTGCATTAACGTCAGCTTCATTAGCCAACCCAACCATAATAGAGAATCCAGAGTTCCTTCGTTTGCGTGAGGAAGAAAAGGAGAAAGCAAAAGAAGCAAACAGTGAACAGAGTCGAAGACCAGCACCCCCCACCCCACAACGAGCACCAATCAACAAACAGATTGAGACGAGGACAGCAGATGGGAAAAGAAGAATCACACCAATGTTTATTCCTCCAGCAGAAGGTTGCAGGACTGAAAATCCAAATAAATCATATGAGGCCAGCTTCTCGTCCAGTCAACAAGAGAAGAGCAAGATAGTAGTTGAGAAAGTGGATGGGGTGGTGGAGCCCAACGTCACCAGTCGACCCCCTCTGGCATTCAATTCAAACACAAACACATCCCCAAGCAAGGGAGCATCAGCTCCTCTCCCTGCTGAATTCAATGGGGTTCCTGCAGGTCTGCCTGCAAGATTTGAAAAATCAACTAATGGTTCAGAAAAACAAATGGATGGACAGATTACAACAATCTCAAGTAAGCGCAAGCATGAAGATGCCCAGCCTATTATGGTCAAGCGCAAACGTGGCAGACCACCCTTATATGAAAGAGCTGCAGCCCCACCACCGAACCGTGTTCCCACTCCACCTCCTGCACAGCCAGTGGTATCTTCAAGACCACAGGCTGTTTCACACACACAGGGAGAGCCTTTAAATGTTCTACCTGAAGCATCACTCTCTAAGTCTGCCAGAGTTCAGATTCCCGGAGAGAATTCAACAGATGGTCCCCCTCCACTTCTGATTGTAGAAAACGATTATCGAATTGGGAATCGTGTTGGTATGGGTGGTGGTACACCAAGTCTACACAGAGTCAGCAGAACACAGCTAAATGTTGTGAGTTGGGTGGCTTACCTTACATCTCAAGTCACTGCAGTTGTAGCTACCACAGATGCTGCTGCACTAACCTGTCATGACAAATCACTCCACATATTTGATATAAAGACAGGGGAAATGATGTTACCCCCAGTACAGCTTCAAGGAAAGGCATCACAGCTTTTCGTCCATGAGAACTGCTTGATGGTTATAACAACAGAAGCCACTCTCACAATCTGGGACATCAGTAAGAAGAAGCGAATTCTCTCAGAGACCATTCGACATTTAGTTGAAGGGCGTAACGGCGGAGTGAAGGCTGTCCAGCTCACTGCTGAAGGTACACCAGTTGTACTGCTTGCATCAGGAGAAGCCTTCACCAGATGCCATGACCTGGAGGCTTGGCTTTTGTTAGGTGATTCTGCCAGTGTACGGCGAGTCCTGCAGCCAGAATACACAATGCTGCCTCCCTGTCATCAAAGTGAATATAAAGCAAGACCTCTTGCTAGTCTCTTGCACCAGACTGCTGCCTATTCTGGCTCTTCAAGAGTGACAAGACCTCTGATCCCAGATGATGCCAAGCAGAACTTGACTGAGGTCTTCATCAACCGCATGCTGACATCTGCCCAGTATCTCAGAGCAGCCCAGGATTACAAATTTTGGTTGAAACGTAAAATTCAATTCTTCATAAAAGAGGGGCTTGAAAAGAAATTGCGAGATGTATTTGATGACCTTCTGGGACCGAGACATAGTGGATCTATGGACAATCAAGATCAAAATGGATGGATGCCCAAGATCATGGACTTTGATAAAAAGAGTCTGCTTGAAGAGTTCCTTCGAGAGATTCTGCTTGCTGAGAACTCCTTGCAGCTACAGAGGCTCTATTCTGAGTATAAGGAACAGCTGGAAGGCAACCTTATAGACACTATTTAATGTAGCATACTTGGGCATACAATTTTGATATAATACTGATATTTCGGGAAGTACTGTAATCTTTATCTCGTGACCAATATCTTGAATTTAGATGCTGTTAGTGCTTATATGTCTTTAGTTGAATTTTAGCCATGGATTTTGTCGTCATGAGTTGTAGAAAGAAATGAAGGCTGACTTCTTATGAGGCACCCCAATATCTTGACAAATTTGGAAATTGATTTTTGTGGATAATTGCTAACCCAAAATCATTACATAAACTTGAGGTAATCAGATAAATAACTCTATGAAGTCAATCTTATTAATTCTACTCCTTTTTAAAAACAGTCCCTGCCTTTTGTATAAATATTGTATTTTAGGGTTTTGTGCATAGTGTTCACATGTGATGACGATAGTAATAAGACTGGCGATTAGAAAGCAGCCATTGCTATATGATAATATACATATTTAAAACTGGTGAATAGCATGAACTAGCAATAAGCCAGCTTAGTTCATGTATTGCTCTATTATGCATATGTTTAACTATAAATTGATGTTATTCAAGACAAAGATCACTGCCAGTGACATCTCTTGCCTTAAACAAGTTTGTAAACTCGATAATACTGGCTTTCTTACATTGAGCCAGATATTATGAGGTTGGCTCTGACTACTCAACTGCCAGAAGAACTGTTTCCACTCACTGCCAGATCATATATATATATAAGAGACCCGGTGTCTGCTACATGGTAGAGCATTAGGTTATCAAATAACTGACCTCGTACCTGTAGCTCCTTACCCTCCCTGATGCACTGCCAGTCGTAAAAATGAACTTAAATTAGAAAGTTGGTACATAGGAAGTGTGGGTATGAATGTTTACAATGATGTAAGTTTACCTGTATAATCATGCAATTAAGTGTGAAACTCATGAAAGTTAGCAAGCATAAATGCTAGTGAATGCTGTACATAAGTATTCATTATATTAATGACAATCTTTTTGTGCAGCAAAAAAAAAAAAAGAAAAAAAAGTCAAGCGCT

**Sanger sequencing, size: 3941**

CAAAGATGGCGGAGGAAGCGTCTTAGCGATTTAGGCGGCGTTTGAATAATGTCTTTGTGTGATTGGAAAGAGACAGAACAAGTATCTTGTTTGTTCTGAAGAGGCTGGAAGTACCATGAAGCTCCTAAAACCGCCTTGGATCAACCATGATGGTGACAAGGCAATCTTCAGTGTTGACCTACATCCTGATGGTTCAAGATTTGCGACAGGTGGACAAGGACAGGATTCCTCTGGAAGGGTTGTGGTGTGGAACCTGAAACCAGTTGCTTTAGAGAAATATGAGGAAGATGAGAAGGTACCGAAGTTGTTGTGTATGCTAGACCAGCACTTGGGGTGTGTGAATTGTGTGAGGTGGTCTCAGAGTGGACGTTTCTTAGCTTCTGCTGGAGACGATAAAGTTATTATCATCTGGCAGCAGAGCAGTTATGGAGGTGGAGCTGTATTTGGTTCAAATGTTGTGAATGTTGAATCTTGGCGAAGTGTCACAACACTAAGAGGCCACAATGGGGATATCCTCGATGTAGCTTGGTCTCCAGGGGATGTTTGGCTAGCATCTGCTTCTGTAGACAATAATGTTATTGTATGGAATACTGCAAAATGGCCAGAACAAGTGCAGATTCTTCGTGGACATACAGGATTGGTGAAAGGTGTTACTTGGGATCCTGTTGGTCGTTACTTAGCCTCCCAGGCAGATGACAAAAGTCTAAGGATTTGGCGAACAACTGACTGGACCACAGAAGAAAAAATAATTGACCCTTTCAGTGAGTGTGGTGGCACAACAACAGTCCTTCGGCCAAGCTGGTCACCTGATGGTCAGTATCTGGTCTCTGCACATGCAATGAACAATGGAGGTCCTACTGCACAGATTGTAGAAAGAGATGGATGGAACACTGACAAGGATTTTGTTGGCCATCGTAAACCTATCACGTGTGTGAGGTTTAATCCTAATATCCTGGAGAAAGCTGATCCAAAGACAGGGAAGTCTGTGCAGTACTGTTGTGTGGCAATTGGCTCCAGGGATCGCTCAATCTCTATTTGGTTTACATCGCTGAAGAGACCTTTGGTGGTGATTCATGATATATTTGATGACTCAGTTCTAGATCTTTCATGGAGCAGTTGTGGCAAGTATCTCATGGCAGTGTCAAAAGATGGTTCTTTGGCCTTCATAAACTTTTCCCCAGATGAGATTGGCCGTCCACTTTGTGAAGATGAAACTAATAACCTCCACATGAAGTTGTACGGTAAATCTGCATTAACGTCAGCTTCATTAGCCAACCCAACCATAATAGAGAATCCAGAGTTCCTTCGTTTGCGTGAGGAAGAAAAGGAGAAAGCAAAAGAAGCAAACAGTGAACAGAGTCGAAGACCAGCACCCCCCACCCCACAACGAGCACCAATCAACAAACAGATTGAGACGAGGACAGCAGATGGGAAAAGAAGAATCACACCAATGTTTATTCCTCCAGCAGAAGGTTGCAGGACTGAAAATCCAAATAAATCATATGAGGCCAGCTTCTCGTCCAGTCAACAAGAGAAGAGCAAGATAGTAGTTGAGAAAGTGGATGGGGTGGTGGAGCCCAACGTCACCAGTCGACCCCCTCTGGCATTCAATTCAAACACAAACACATCCCCAAGCAAGGGAGCATCAGCTCCTCTCCCTGCTGAATTCAATGGGGTTCCTGCAGGTCTGCCTGCAAGATTTGAAAAATCAACTAATGGTTCAGAAAAACAAATGGATGGACAGATTACAACAATCTCAAGTAAGCGCAAGCATGAAGATGCCCAGCCTATTATGGTCAAGCGCAAACGTGGCAGACCACCCTTATATGAAAGAGCTGCAGCCCCACCACCGAACCGTGTTCCCACTCCACCTCCTGCACAGCCAGTGGTATCTTCAAGACCACAGGCTGTTTCACACACACAGGGAGAGCCTTTAAATGTTCTACCTGAAGCATCACTCTCTAAGTCTGCCAGAGTTCAGATTCCCGGAGAGAATTCAACAGATGGTCCCCCTCCACTTCTGATTGTAGAAAACGATTATCGAATTGGGAATCGTGTTGGTATGGGTGGTGGTACACCAAGTCTACACAGAGTCAGCAGAACACAGCTAAATGTTGTGAGTTGGGTGGCTTACCTTACATCTCAAGTCACTGCAGTTGTAGCTACCACAGATGCTGCTGCACTAACCTGTCATGACAAATCACTCCACATATTTGATATAAAGACAGGGGAAATGATGTTACCCCCAGTACAGCTTCAAGGAAAGGCATCACAGCTTTTCGTCCATGAGAACTGCTTGATGGTTATAACAACAGAAGCCACTCTCACAATCTGGGACATCAGTAAGAAGAAGCGAATTCTCTCAGAGACCATTCGACATTTAGTTGAAGGGCGTAACGGCGGAGTGAAGGCTGTCCAGCTCACTGCTGAAGGTACACCAGTTGTACTGCTTGCATCAGGAGAAGCCTTCACCAGATGCCATGACCTGGAGGCTTGGCTTTTGTTAGGTGATTCTGCCAGTGTACGGCGAGTCCTGCAGCCAGAATACACAATGCTGCCTCCCTGTCATCAAAGTGAATATAAAGCAAGACCTCTTGCTAGTCTCTTGCACCAGACTGCTGCCTATTCTGGCTCTTCAAGAGTGACAAGACCTCTGATCCCAGATGATGCCAAGCAGAACTTGACTGAGGTCTTCATCAACCGCATGCTGACATCTGCCCAGTATCTCAGAGCAGCCCAGGATTACAAATTTTGGTTGAAACGTAAAATTCAATTCTTCATAAAAGAGGGGCTTGAAAAGAAATTGCGAGATGTATTTGATGACCTTCTGGGACCGAGACATAGTGGATCTATGGACAATCAAGATCAAAATGGATGGATGCCCAAGATCATGGACTTTGATAAAAAGAGTCTGCTTGAAGAGTTCCTTCGAGAGATTCTGCTTGCTGAGAACTCCTTGCAGCTACAGAGGCTCTATTCTGAGTATAAGGAACAGCTGGAAGGCAACCTTATAGACACTATTTAATGTAGCATACTTGGGCATACAATTTTGATATAATACTGATATTTCGGGAAGTACTGTAATCTTTATCTCGTGACCAATATCTTGAATTTAGATGCTGTTAGTGCTTATATGTCTTTAGTTGAATTTTAGCCATGGATTTTGTCGTCATGAGTTGTAGAAAGAAATGAAGGCTGACTTCTTATGAGGCACCCCAATATCTTGACAAATTTGGAAATTGATTTTTGTGGATAATTGCTAACCCAAAATCATTACATAAACTTGAGGTAATCAGATAAATAACTCTATGAAGTCAATCTTATTAATTCTACTCCTTTTTAAAAACAGTCCCTGCCTTTTGTATAAATATTGTATTTTAGGGTTTTGTGCATAGTGTTCACATGTGATGACGATAGTAATAAGACTGGCGATTAGAAAGCAGCCATTGCTATATGATAATATACATATTTAAAACTGGTGAATAGCATGAACTAGCAATAAGCCAGCTTAGTTCATGTATTGCTCTATTATGCATATGTTTAACTATAAATTGATGTTATTCAAGACAAAGATCACTGCCAGTGACATCTCTTGCCTTAAACAAGTTTGTAAACTCGATAATACTGGCTTTCTTACATTGAGCCAGATATTATGAGGTTGGCTCTGACTACTCAACTGCCAGAAGAACTGTTTCCACTCACTGCCAGATCATATATATATATAAGAGACCCGGTGTCTGCTACATGGTAGAGCATTAGGTTATCAAATAACTGACCTCGTACCTGTAGCTCCTTACCCTCCCTGATGCACTGCCAGTCGTAAAAATGAACTTAAATTAGAAAGTTGGTACATAGGAAGTGTGGGTATGAATGTTTACAATGATGTAAGTTTACCTGTATAATCATGCAATTAAGTGTGAAACTCATGAAAGTTAGCAAGC
